# Shuttling Tolerogenic Dendritic Cells across the Blood–Brain Barrier *In Vitro via* the Introduction of *De Novo* C–C Chemokine Receptor 5 Expression Using Messenger RNA Electroporation

**DOI:** 10.3389/fimmu.2017.01964

**Published:** 2018-01-23

**Authors:** Maxime De Laere, Judith Derdelinckx, Mari Hassi, Mari Kerosalo, Heidi Oravamäki, Johan Van den Bergh, Zwi Berneman, Nathalie Cools

**Affiliations:** ^1^Laboratory of Experimental Hematology, Faculty of Medicine and Health Sciences, Vaccine & Infectious Disease Institute (VAXINFECTIO), University of Antwerp, Wilrijk, Belgium; ^2^Department of Neurology, Antwerp University Hospital, Edegem, Belgium; ^3^Center for Cell Therapy and Regenerative Medicine, Antwerp University Hospital, Edegem, Belgium

**Keywords:** tolerogenic dendritic cells, C–C chemokine receptor 5, messenger RNA electroporation, migration, blood–brain barrier, multiple sclerosis

## Abstract

The use of tolerance-inducing dendritic cells (tolDCs) has been proven to be safe and well tolerated in the treatment of autoimmune diseases. Nevertheless, several challenges remain, including finding ways to facilitate the migration of cell therapeutic products to lymph nodes, and the site of inflammation. In the treatment of neuroinflammatory diseases, such as multiple sclerosis (MS), the blood–brain barrier (BBB) represents a major obstacle to the delivery of therapeutic agents to the inflamed central nervous system (CNS). As it was previously demonstrated that C–C chemokine receptor 5 (CCR5) may be involved in inflammatory migration of DCs, the aim of this study was to investigate CCR5-driven migration of tolDCs. Only a minority of *in vitro* generated vitamin D_3_ (vitD_3_)-treated tolDCs expressed the inflammatory chemokine receptor CCR5. Thus, messenger RNA (mRNA) encoding CCR5 was introduced by means of electroporation (EP). After mRNA EP, tolDCs transiently displayed increased levels of CCR5 protein expression. Accordingly, the capacity of mRNA electroporated tolDCs to transmigrate toward a chemokine gradient in an *in vitro* model of the BBB improved significantly. Neither the tolerogenic phenotype nor the T cell-stimulatory function of tolDCs was affected by mRNA EP. EP of tolDCs with mRNA encoding CCR5 enabled these cells to migrate to inflammatory sites. The approach used herein has important implications for the treatment of MS. Using this approach, tolDCs actively shuttle across the BBB, allowing *in situ* down-modulation of autoimmune responses in the CNS.

## Introduction

Multiple sclerosis (MS) is a chronic autoinflammatory disease of the central nervous system (CNS), mediated by myelin-reactive T cells that escape central and peripheral tolerance mechanisms and induce inflammation and tissue damage within the CNS (1, 2). During the last two decades, several new and increasingly efficacious therapeutics have become available for the treatment of MS (3). However, this higher treatment efficacy is associated with a more hazardous adverse event profile (4), and none of the currently approved treatments is successful in completely halting MS. In addition, as the disease progresses, these therapeutics become less effective. The blood–brain barrier (BBB) represents a major hurdle in the treatment of this neuroinflammatory disorder. Previous authors hypothesized that during the progressive phases of MS, inflammation is trapped behind an intact BBB and hence is not accessible to immunomodulatory agents (5–7). Finding ways to improve the access of therapeutic agents to the CNS would undoubtedly and markedly improve the treatment outcome in progressive forms of MS.

Major advancements in current knowledge of immunology, together with increased understanding of the processes underlying MS and mechanisms contributing to immune tolerance, have led to the emergence of immune-regulatory cell therapy as a promising strategy to restore tolerance in MS (8, 9). Tolerance-inducing dendritic cells (tolDCs) or tolerogenic dendritic cells have a unique ability to steer the host immune response toward tolerance induction (10). In general, tolDCs can be defined as maturation-resistant DCs, characterized by low to intermediate expression levels of major histocompatibility complex (MHC) class II and costimulatory molecules (11). They mediate tolerance by inducing T-cell anergy, deleting autoreactive T cells, and/or inducing and expanding the population of regulatory T cells (11). In early clinical trials, tolDC-based therapies were proven to be safe and well tolerated for the treatment of autoimmune diseases (12–15). The efficacy of this treatment approach remains to be determined in further clinical studies, and factors that affect the efficacy of tolDC-based therapies are not yet fully understood.

The migratory capacity of tolDCs may influence the potential clinical use of these cells. It can be reasoned that *in vivo* efficacy of tolDC-based therapies will depend not only on their potency (i.e., ability to induce tolerance) but also on their probability of encountering T cells and thus their ability to reach target organs (i.e., lymph nodes and CNS) in MS. DC migration to lymph nodes is mainly determined by C-C chemokine receptor 7 (CCR7) (16). CCR5, on the other hand, is a key molecule involved in guiding DCs to the site of inflammation (17). Some studies reported that expression levels of the CCR5 ligands CCL3, CCL4, and CCL5 were upregulated in lesions and cerebrospinal fluid of patients with MS (18–22). We (23) and others (24) demonstrated that circulating DCs of MS patients expressed increased levels of CCR5. Based on these findings, we hypothesized that the expression of CCR5 on tolDCs might drive DC migration to an inflamed CNS.

In animal model studies, the presence of steady-state or tolerogenic DCs in the CNS suppressed experimental autoimmune encephalomyelitis (EAE) (25–27). Mechanisms underlying this tolerance induction included preferential secretion by DCs of the immunomodulatory cytokines interleukin-10 (IL-10) and transforming growth factor-β, in addition to skewing of the T-cell response by favoring the development of T-helper 2 cells and regulatory T cells, while restraining T-helper 17 cell development. In these studies, DCs were either cultured *in vitro* and injected intracerebrally (27), rendered tolerogenic in the CNS *in situ* by hepatocyte growth factor selectively overexpressed by neurons (25), or implicated in the induction of tolerance after intravenous injection of an autoantigen peptide of myelin oligodendrocyte glycoprotein (26). Previously, we reported a culture protocol for the generation of vitamin D_3_ (vitD_3_)-treated tolDCs (28). Our data showed that vitD_3_-treated tolDCs of MS patients displayed a semi-mature phenotype and an anti-inflammatory cytokine profile. In addition, vitD_3_-treated tolDCs induced antigen-specific T-cell hyporesponsiveness, supporting the clinical potential of these cells in correcting the immunological imbalance inherent in MS. However, it remains to be determined to what extent *in vitro*-generated tolDCs migrate to an inflamed CNS, especially as this requires transmigration across the BBB. Unger et al. (29) reported that tolDCs downregulated CCR5 expression upon proinflammatory stimulation, suggesting that inflammatory trafficking of these cells might be suboptimal. This prompted us to study the CCR5-driven migratory capacity of tolDCs *in vitro* in a previously optimized and characterized model of the BBB (30). We hypothesized that the CCR5-driven migratory capacity of these cells could be optimized by introducing CCR5 protein expression using messenger RNA (mRNA) electroporation (EP). Ultimately, endowing tolDCs with the capacity to migrate to an inflamed CNS by introducing *de novo* CCR5 protein expression will allow optimal exploitation of their tolerogenic capacity. Active shuttling of cells across the BBB would allow for targeted *in situ* down-modulation of autoimmune responses by tolDCs.

## Materials and Methods

### *In Vitro* Generation of Monocyte-Derived Dendritic Cells

Peripheral blood from healthy donors was obtained from buffy coats provided by the Red Cross donor center (Red Cross-Flanders, Mechelen, Belgium). Peripheral blood mononuclear cells were isolated by density gradient centrifugation (Ficoll Pacque PLUS, GE Healthcare, Amsterdam, the Netherlands). From the peripheral blood mononuclear cell fraction, monocytes were purified by CD14^+^ immunomagnetic selection (CD14 Reagent, Miltenyi Biotec, Bergisch Gladbach, Germany), according to the manufacturer’s instructions. The CD14-depleted cell fraction [i.e., peripheral blood lymphocytes (PBLs)] was cryopreserved in fetal bovine serum (Thermo Fisher Scientific, Erembodegem, Belgium) supplemented with 10% dimethyl sulfoxide (Sigma-Aldrich, Bornem, Belgium) and stored at −80°C for later use in an allogeneic mixed leukocyte reaction. CD14^+^ monocytes were cultured *in vitro* at a density of 1–1.2 × 10^6^/ml and differentiated into DCs in culture medium consisting of Iscove’s modified Dulbecco’s medium (IMDM) with l-glutamine (Thermo Fisher Scientific), supplemented with 200 IU/ml of granulocyte-macrophage colony-stimulating factor (Gentaur, Brussels, Belgium), 250 IU/ml of IL-4 (Miltenyi Biotec), 2% human AB (hAB) serum (Thermo Fisher Scientific), 10 µg/ml of gentamicin (Thermo Fisher Scientific), and 1 µg/ml of amphotericin B (Thermo Fisher Scientific). TolDCs were differentiated under the same conditions, except for the addition of 2 nM 1,25(OH)_2_-vitamin D_3_ (vitD_3_, Calcijex, Abbott Laboratories, IL, USA) to the culture medium. On day 4 of culture, DCs were subjected to a proinflammatory cytokine cocktail by the addition of 1,000 IU/ml of IL-1β (Miltenyi Biotec), 1,000 IU/ml of tumor necrosis factor-α (Miltenyi Biotec), and 2.5 µg/ml of prostaglandin E_2_ (Pfizer, Elsene, Belgium) to obtain mature control and tolDCs. For tolDC cultures, vitD_3_ was replenished on day 4. The cells were cultured in a humidified atmosphere with 5% CO_2_ at 37°C. On day 6, DCs were harvested for use in further experiments.

The study was approved by the ethics committee of Antwerp University Hospital and the University of Antwerp (15/50/543) and followed the tenets of the Declaration of Helsinki.

### Messenger RNA EP

The complementary DNA sequence of human *CCR5* (accession number U54994) was modified for optimal codon use (Figure S1 in Supplementary Material) and subcloned into a pST1-plasmid vector under the control of a T7 promotor and with the addition of a poly(A)tail (GeneArt, Thermo Fisher Scientific). After transformation in *Escherichia coli* and linearization of the circular DNA plasmid, mRNA transcripts were generated using a T7 *in vitro* transcription kit (mMessage mMachine T7 kit, Ambion, Life Technologies), according to the manufacturer’s protocol. mRNA was resuspended at a concentration of 1 µg/µl, aliquoted, and stored at −20°C.

Messenger RNA EP of DCs was performed as previously described (31, 32). In brief, the cells were resuspended in Opti-MEM (Thermo Fisher Scientific), and a 200 µL aliquot of this cell suspension containing 2–10 × 10^6^ cells was transferred into a 0.4-cm cuvette (Immunosource, Schilde, Belgium). Next, 10 µg of mRNA were added. EP was performed using a Gene Pulser Xcell™ electroporation system (Bio-Rad, Temse, Belgium) with a time constant protocol at 300 V for 7 ms. EP of cells without the addition of mRNA (mock EP) was performed as a control. Immediately after EP, the cells were transferred into fresh DC culture medium. For tolDCs, 2 nM vitD_3_ was added to the cell culture medium. After a 30-min resting phase, the cells were washed and resuspended in warm IMDM supplemented with 5% hAB serum. Following an additional resting period of 90 min, the cells were washed again, resuspended in IMDM supplemented with 1% hAB serum, and used in further experiments.

### Flow Cytometric Phenotyping

Flow cytometric analysis of the expression of CCR5 by DCs was performed 2, 4, 24, 48, and 72 h after EP. *CCR5* mRNA-electroporated, mock-electroporated, and nonelectroporated DCs were stained with a phycoerythrin-cyanin 7-labeled anti-CCR5-antibody (BD Pharmingen, Erembodegem, Belgium) or an isotype-matched control antibody (BD Pharmingen). LIVE/DEAD^®^ Fixable Violet Dead Cell Stain (Thermo Fisher Scientific) was added to assess cell viability. The indicated percentages of CCR5-positive cells were within the living DC population (i.e., gated for DCs based on light scatter properties and negative for LIVE/DEAD^®^ Fixable Violet Dead Cell staining). Flow cytometric measurements were performed using a Cyflow ML flow cytometer (Partec, Münster, Germany). The results were analyzed using FlowJo software (Tree Star, Ashland, OR, USA).

The phenotype of DCs was characterized using the following fluorochrome-labeled mouse antihuman monoclonal antibodies: anti-CD83-fluorescein isothiocyanate (Life Technologies), anti-CD80-phycoerythrin (BD Pharmingen), antihuman leukocyte antigen (HLA)-DR-peridinin chlorophyll (BD Biosciences), anti-CD86-fluorescein isothiocyanate (BD Pharmingen), and anti-CCR5-phycoerythrin-cyanin 7. Isotype-matched control monoclonal antibodies were used to determine nonspecific background staining. For analytical flow cytometry, at least 10^4^ events were acquired using a FACScan flow cytometer (BD). The indicated percentages were within the DC population based on light scatter properties. All the results were analyzed using FlowJo software.

### *In Vitro* BBB Model

The *in vitro* BBB model was constructed as described previously (30). In brief, human primary astrocytes (Sanbio, Uden, the Netherlands) were seeded at a density of 15,000 cells/cm^2^ on the poly-l-lysine-coated underside of a transwell (24-well format) with 3.0-µm pore size (Greiner Bio-one, Vilvoorde, Belgium) and allowed to adhere for 2 h. Subsequently, the inserts were transferred into a well filled with EGM-2-MV medium (Lonza, Verviers, Belgium) with 2.5% fetal bovine serum. hCMEC/D3 endothelial cells (Tébu-bio, Le Perray-en-Yvelines, France) were seeded onto the insert’s collagen-coated upper side at a density of 25,000 cells/cm^2^. Cultures were maintained in EGM-2-MV medium in 5% CO_2_ at 37°C. Three days after initiating the coculture, the growth medium was replaced by EBM-2-plus medium, consisting of EBM-2 medium (Lonza), supplemented with 1.4 µM hydrocortisone (Pfizer), 1 ng/ml of basic fibroblast growth factor (Thermo Fisher Scientific), 10 µg/ml of gentamicin, 1 µg/ml of amphotericin-B, and 2.5% fetal bovine serum. EBM-2-plus medium was replenished every other day. Migration assays were performed between days 10 and 13 of culture.

### Migration Assay

Chemotaxis of DCs was studied 2 h after EP or at an equivalent time point for nonelectroporated DCs using 3.0-μm-sized pore transwells and an *in vitro* BBB model. DCs (2 × 10^5^) were added to the upper compartment of both the transwell and *in vitro* BBB model. The basolateral compartment contained 25 ng/ml of CCL4 and 25 ng/ml of CCL5 in IMDM, supplemented with 1% hAB serum. DCs were subsequently allowed to migrate for 4 h in the transwell assays or for 24 h in assays using the *in vitro* BBB model. The negative control consisted of 2 × 10^5^ DCs added to the upper compartment, while no chemokines were added to the basolateral compartment. As a positive control, 2 × 10^5^ DCs were added directly to the basolateral compartment. At the indicated time points, DCs were collected from the basolateral compartment. After resuspension in a fixed volume of 200 µl, they were counted using a BD FACScan flow cytometer. Events were acquired at a fixed flow rate for exactly 120 s. The results were analyzed using FlowJo software. The percentage migration was calculated as follows:

$$\left[\begin{array}{l} \left(\text{\# migrated cells in the experimental sample}-\text{\# migrated cells in the negative control}\right)/\\ \text{\# cells in the positive control} \end{array}\right]*100\%.$$

### RNA Isolation and Quantitative Real-time Polymerase Chain Reaction (qPCR)

For analysis of the gene expression profile of nonelectroporated and electroporated tolDCs and control DCs, total RNA was isolated. The cells were disrupted and homogenized using guanidine-thiocyanate-containing lysis buffer. Total RNA was isolated using an RNeasy microkit (Qiagen, Antwerp, Belgium). The RNA concentration was determined by measuring absorbance at 260 nm using a Nanodrop spectrophotometer (Wilmington, DE, USA). Reverse transcription of the obtained RNA into cDNA was performed using an iScript™ Advanced cDNA Synthesis Kit (Bio-Rad). Subsequently, SYBR^®^ Green technology was used for relative mRNA quantification by qPCR in a CFX96 C1000 thermal cycler (Bio-Rad). qPCR reactions were conducted at 95°C for 30 s, followed by 40 cycles at 95°C for 5 s, and at 60°C for 30 s. All primer sets were obtained from Bio-Rad; validation data are shown in Table S1 in Supplementary Material. qPCR was performed in triplicate, and resulting mRNA levels were normalized to levels of the reference genes beta-actin and phosphoglycerate kinase 1. Melt curve analysis was performed to confirm the specificity of the amplified product. Bio-Rad CFX manager v3.1 was used for data processing and analysis.

### Allogeneic Mixed Lymphocyte Reaction

To assess the allogeneic T-cell stimulatory capacity of DCs, the cells were cocultured with allogeneic PBLs in a 1:10 ratio. Nonstimulated responder PBLs served as a negative control, and allogeneic PBLs stimulated with 1 µg/ml of phytoheamagglutinin (Sigma-Aldrich) were used as a positive control. Cocultures were performed in IMDM supplemented with 5% hAB serum at 37°C. After 6 days in coculture, the secreted level of interferon-γ (IFN-γ) in the cell culture supernatant was determined in duplicate as a measure of allo-stimulatory capacity using a commercially available enzyme-linked immunosorbent assay (ELISA) kit (PeproTech, NJ, USA). In addition, IL-10 secretion was measured in the supernatant using a U-PLEX assay (Meso Scale Discovery, MD, USA), according to the manufacturer’s instructions.

### Statistical Analysis

Data were analyzed using Graphpad Prism software version 5.01 (Graphpad, San Diego, CA, USA), except for qPCR data, which were analyzed using CFX Manager software, version 3.1 (Bio-Rad). Comparison of nonelectroporated, mock-electroporated, and *CCR5* mRNA-electroporated tolDCs was performed by a repeated measures one-way ANOVA, followed by Tukey’s multiple comparisons test. For data that were not normally distributed according to the Kolmogorov–Smirnov test, the Friedman test, with Dunn’s multiple comparison test was performed. Comparison of CCR5 expression levels at several time points after EP in mock-electroporated, *CCR5* mRNA-electroporated, and nonelectroporated tolDCs was performed using a two-way repeated measures ANOVA, with *post hoc* Bonferroni tests. For qPCR results, differences were considered significant when *p* < 0.01. For other data, statistical significance was considered at the 5% level. Data are shown as mean ± SEM. The number of biological replicates is indicated in the figure or table legend.

## Results

### TolDCs Displayed Limited CCR5-Driven Migratory Capacity

Only a minority of *in vitro* generated vitD_3_-treated tolDCs expressed CCR5 (i.e., 12.96 ± 2.02% on average). This translated into marginal chemotaxis of tolDCs. Only 0.10 ± 0.05% of tolDCs migrated in response to the chemokines CCL4 and CCL5.

### mRNA EP Resulted in a Marked Increase of CCR5 Expression in tolDCs

**Figure 1:**
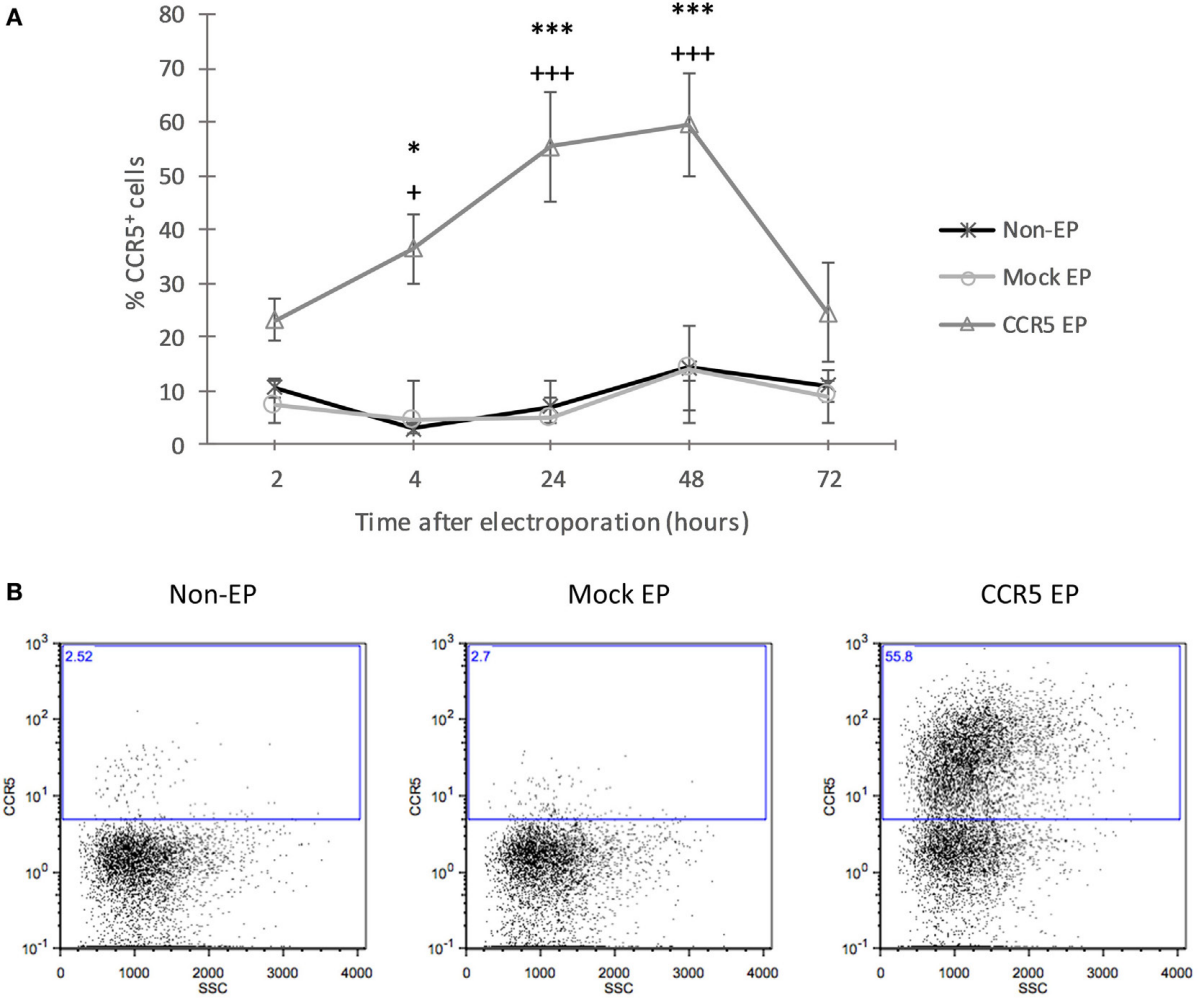
Transfection with messenger RNA (mRNA) encoding C–C chemokine receptor 5 (CCR5) using electroporation (EP) resulted in a transient increase of CCR5 protein expression by tolerance-inducing dendritic cells (tolDCs). **(A)** The protein expression level of CCR5 showed an incremental increase in CCR5 mRNA-electroporated tolDCs from 2 to 48 h after EP, and the expression of CCR5 declined 72 h after EP (mean ± SEM of five replicates) ^+^Denotes a statistically significant difference from mock EP, *Denotes a statistically significant difference from non-EP, ^*/+^*p* < 0.05, ^**/++^*p* < 0.01, ^***/+++^*p* < 0.001. **(B)** Representative dot plots displaying CCR5 expression by nonelectroporated, mock-electroporated, and *CCR5* mRNA-electroporated tolDCs, as assessed by flow cytometry 24 h after EP.

To increase CCR5 protein expression, tolDCs were electroporated with mRNA encoding CCR5. Using flow cytometric analysis of CCR5 protein expression at consecutive time points after EP, an incremental increase was detected in CCR5 expression levels from 2 to 48 h following EP, after which expression decreased again (Figure 1). CCR5 expression levels of *CCR5* mRNA-electroporated DCs were significantly higher as compared with those of both nonelectroporated and mock-electroporated DCs 4 h (36.84 ± 5.89 vs. 2.87 ± 0.60 and 4.75 ± 0.79%, respectively; *p* < 0.05), 24 h (55.42 ± 10.09 vs. 7.09 ± 3.04 and 5.04 ± 1.74%, respectively; *p* < 0.001), and 48 h (59.52 ± 9.59 vs. 14.44 ± 9.48 and 14.08 ± 7.86%, respectively; *p* < 0.001) after EP.

### CCR5 mRNA-Electroporated tolDCs Demonstrated Increased CCR5-Driven Migration *In Vitro*

**Figure 2:**
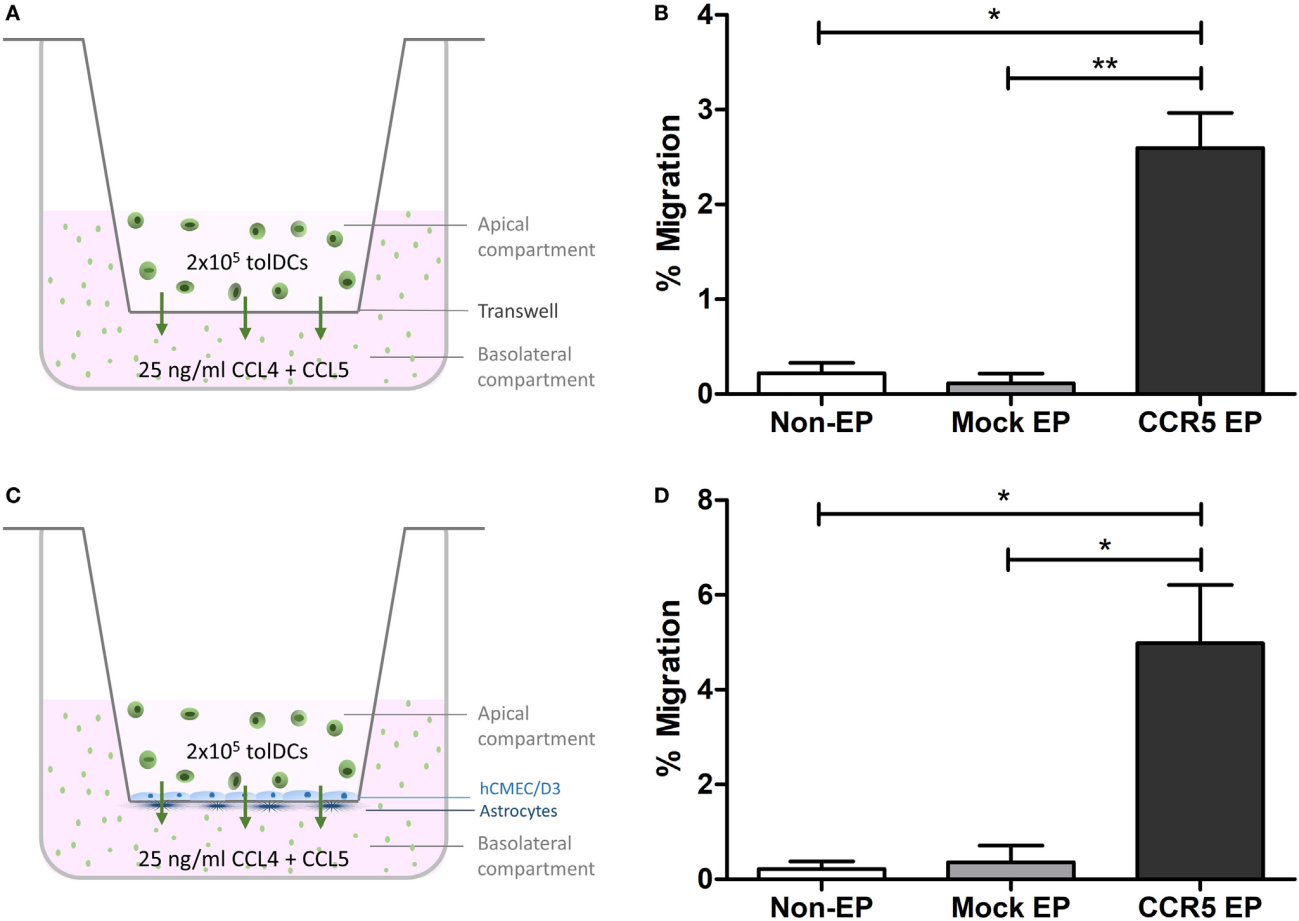
*In vitro* C–C chemokine receptor 5 (CCR5)-driven migration was increased following *CCR5* messenger RNA (mRNA) electroporation (EP). **(A)** Schematic overview of the transwell migration experiment. **(B)** *CCR5* mRNA-electroporated tolerance-inducing dendritic cells (tolDCs) showed enhanced migratory capacity toward CCR5 ligands CCL4 and CCL5 in a transwell chemotaxis assay (mean ± SEM of seven replicates). *p < 0.05, **p < 0.01, ***p < 0.001. **(C)** Schematic overview of the tolDC migration experiment using an *in vitro* blood–brain barrier (BBB) model. **(D)** Although nonelectroporated and mock-electroporated tolDCs displayed only a limited capacity to transmigrate through the BBB *in vitro* in response to CCL4 and CCL5, EP of tolDCs with *CCR5* mRNA increased their transmigratory capacity in response to chemokines added basolaterally in the *in vitro* BBB model (mean ± SEM of six replicates). *p < 0.05, **p < 0.01, ***p < 0.001.

To investigate whether elevated CCR5 expression translated into a higher capacity to migrate *in vitro*, chemotaxis of tolDCs across transwells in response to the CCR5 ligands CCL4 and CCL5 was studied. Although only 0.22 ± 0.11% of nonelectroporated tolDCs and 0.11 ± 0.10% of mock-electroporated tolDCs migrated toward a CCL4 and CCL5 gradient, 2.59 ± 0.37% of *CCR5* mRNA-electroporated tolDCs showed chemokine-mediated migration (*p* < 0.05 and *p* < 0.01, respectively) (Figures 2A,B). Likewise, there was an 18-fold increase in CCR5-driven transmigration of tolDCs across an *in vitro* BBB model following *CCR5* mRNA EP of tolDCs. Only 0.22 ± 0.16% of nonelectroporated tolDCs and 0.35 ± 0.35% of mock-electroporated tolDCs succeeded in transmigrating across the *in vitro* BBB model. In contrast, 4.98 ± 1.24% of tolDCs electroporated with *CCR5* mRNA crossed the BBB in response to CCL4 and CCL5 (*p* < 0.05) (Figures 2C,D).

### mRNA EP Did Not Affect the Tolerogenic Phenotype and Function of tolDCs

To ensure that the semi-mature phenotype of tolDCs was unaffected by mRNA EP, the expression of DC maturation markers, as well as that of molecules involved in antigen presentation, was investigated (Table 1). Mock or mRNA EP did not affect the proportion of control DCs or that of tolDCs expressing CD80, CD83, CD86, and HLA-DR. In addition, the level of protein expression per cell, as assessed by mean fluorescence intensity, was not significantly affected for the membrane molecules expressed by tolDCs following mRNA EP. In control DCs, a modest but significant decrease in protein expression levels was observed after EP for all molecules tested. However, expression levels of CD80, CD83, CD86, and HLA-DR were still significantly higher in *CCR5* mRNA-electroporated control DCs as compared to tolDCs. Interestingly, neither mock nor mRNA EP affected mRNA expression levels of LILRB4 and TLR2, two established regulators of tolerogenicity, in vitD_3_-treated tolDCs (33). Normalized expression levels of both markers remained significantly higher in tolDCs as compared with those of control DCs (Table 1).

**Table 1:** The semi-mature phenotype and tolerogenic messenger RNA (mRNA) expression profile of tolerance-inducing dendritic cells (TolDCs) was not affected by mRNA electroporation.

		Control DCs			TolDCs		
		Non-EP	Mock EP	C–C chemokine receptor 5 (CCR5) EP	Non-EP	Mock EP	CCR5 EP
**Protein expression**							
CD80	%	92.45 ± 1.69	87.97 ± 2.85	86.13 ± 3.92	20.18 ± 9.85***	16.45 ± 8.01***	16.37 ± 8.33***
	MFI	120.47 ± 12.09	81.15 ± 6.44^+++^	78.28 ± 6.81^+++^	40.60 ± 5.98***	39.35 ± 5.16***	38.23 ± 5.26***
CD83	%	90.95 ± 1.15	82.58 ± 2.22	82.55 ± 2.65	24.83 ± 7.56***	23.48 ± 6.58***	22.22 ± 6.90***
	MFI	29.50 ± 2.90	22.88 ± 3.01^+++^	23.55 ± 3.28^++^	16.48 ± 3.59***	15.67 ± 3.32***	15.65 ± 3.40***
CD86	%	99.33 ± 0.26	99.20 ± 0.30	99.10 ± 0.36	93.03 ± 2.01**	92.73 ± 2.05**	93.52 ± 2.13**
	MFI	762.33 ± 55.37	508.50 ± 82.98^++^	471.67 ± 8,797^+++^	212.65 ± 44.15***	165.17 ± 36.28***	183.30 ± 40.67***
HLA-DR	%	93.87 ± 5.85	92.95 ± 6.39	93.07 ± 6.43	88.82 ± 5.18*	88.02 ± 5.65	87.87 ± 5.64*
	MFI	337.00 ± 58.42	220.33 ± 25.92^+^	234.33 ± 21.40	97.05 ± 34.91***	75.95 ± 19.53**	75.07 ± 21.63**

**mRNA expression**							
LILRB4	RNE	0.28570 ± 0.01755	0.29916 ± 0.00928	0.25645 ± 0.01017	1.90876 ± 0.11275***	1.95846 ± 0.196215***	1.96215 ± 0.2019***
TLR2	RNE	0.10107 ± 0.00992	0.11338 ± 0.00490	0.10679 ± 0.00676	1.74427 ± 0.15793***	1.95846 ± 0.22097***	2.04363 ± 0.27273***

*Mean ± SEM of six replicates (protein expression) and three replicates (mRNA expression)*.

*^+^Denotes a statistically significant difference from nonelectroporated DCs, within control DC or tolDC conditions*.

*Denotes a statistically significant difference from the corresponding (i.e., non-EP, mock EP, or *CCR5* EP) control DC conditions.

^*/+^p < 0.05, ^**/++^p < 0.01, ^***/+++^p < 0.001.

*MFI, mean fluorescence intensity; RNE, relative normalized expression*.

**Figure 3:**
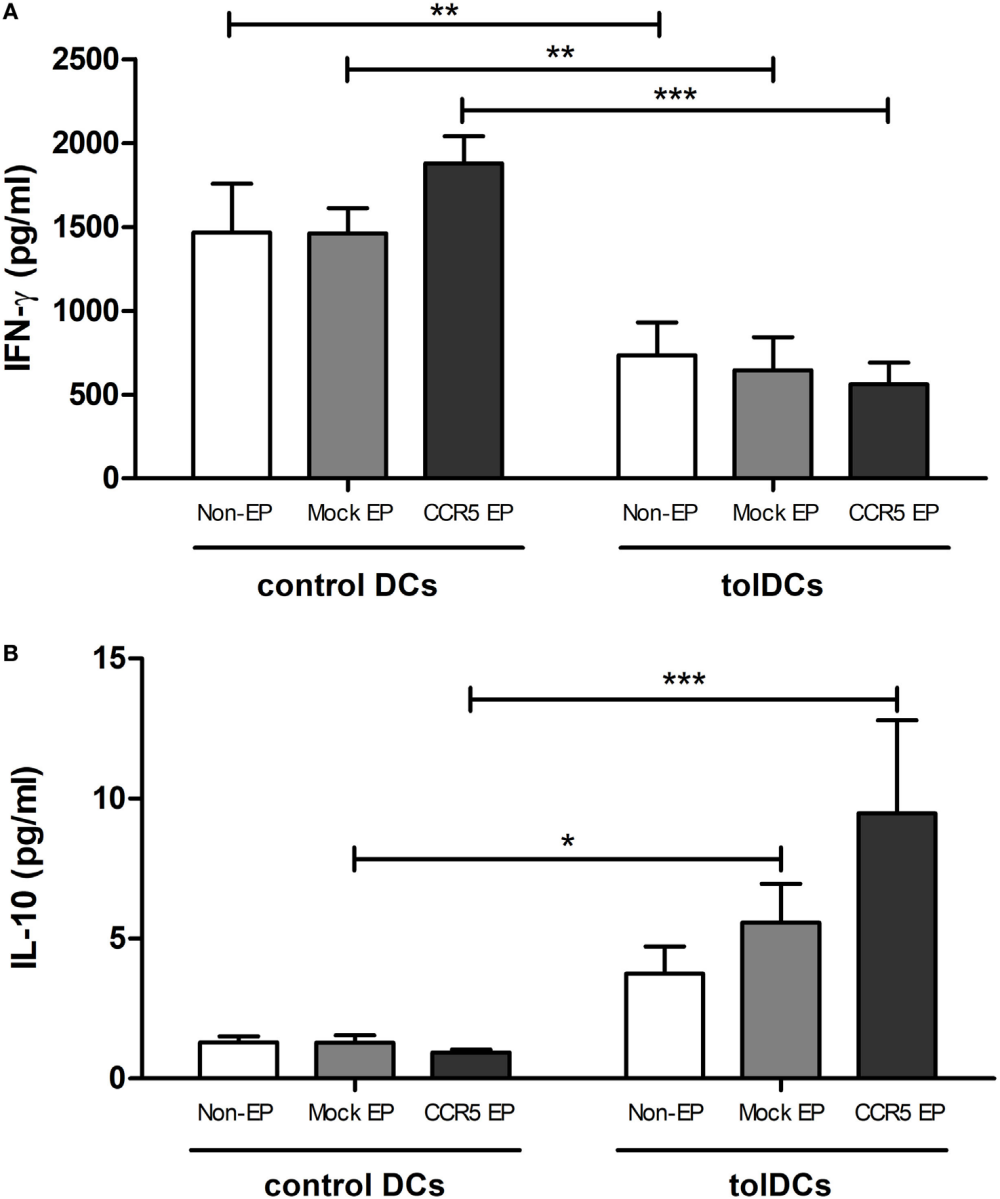
Messenger RNA (mRNA)-electroporated tolerance-inducing dendritic cells (tolDCs) maintained their capacity to induce T-cell hyporesponsiveness while stimulating IL-10 secretion in an allogeneic mixed leukocyte reaction. **(A)** IFN-γ levels in the supernatant of allogeneic peripheral blood lymphocytes (PBLs) cultured in the presence of control DCs or tolDCs were analyzed by an enzyme-linked immunosorbent assay. No differences in the level of secreted IFN-γ were observed in the supernatant of PBLs stimulated with nonelectroporated tolDCs as compared with that of mock- or C–C chemokine receptor 5 (*CCR5*) mRNA-electroporated tolDCs. **(B)** Levels of IL-10 in the supernatant of PBLs cocultured with control or tolDCs. Cocultures of PBLs with mock- or *CCR5* mRNA-electroporated tolDCs contained higher levels of IL-10 as compared with cocultures of PBLs with the corresponding control DCs [mean ± SEM of four replicates (IFN-γ) or six replicates (IL-10)]. *p < 0.05, **p < 0.01, ***p < 0.001.

Functionally, tolDCs maintained their capacity to induce T-cell hyporesponsiveness following mRNA EP (Figure 3A). No differences were observed in the level of secreted IFN-γ in the supernatant of PBLs stimulated with nonelectroporated tolDCs as compared with that of PBLs stimulated with either mock- or *CCR5* mRNA-electroporated tolDCs. In contrast, the levels of IL-10 secreted in the coculture supernatant were significantly higher when mock- or *CCR5* mRNA-electroporated tolDCs were cocultured with PBLs as compared with cocultures of PBLs with corresponding control DCs (*p* < 0.05 and *p* < 0.001, respectively) (Figure 3B).

## Discussion

TolDC-based therapies represent a promising strategy for the future treatment of autoimmune diseases, such as MS. DCs are key players in maintaining the balance between immunity and tolerance by priming T-cell responses in an antigen-specific manner (34). This makes them ideal vehicles for modulating detrimental autoimmune reactions in a disease-specific way, without compromising immune surveillance and host-protective mechanisms. Although previous research confirmed the safety and tolerability of tolDC treatment in patients with type I diabetes (13), rheumatoid arthritis (12, 35, 36), and Crohn’s disease (15), the efficacy of tolDC-based treatments in human autoimmune diseases remains to be determined. In this regard, it can be envisaged that the ability of *in vitro* generated tolDCs to downmodulate an ongoing pathological immune response *in vivo* will critically depend on their ability to reach both secondary lymphoid organs and the site of inflammation. In MS, therapeutic access to the CNS is hindered by the BBB. The aforementioned could explain, at least in part, poor treatment responses typically observed in MS, especially in progressive disease stages, in which the BBB is hypothesized to encapsulate inflammation within the CNS (6, 7).

The BBB is also a major obstacle for tolDCs administered peripherally, making it difficult for them to reach the CNS for *in situ* down-modulation of ongoing inflammation. Following cell tracking and imaging of intravenously administered vitD_3_-treated tolDCs, Mansilla et al. (37) found only a transient and low level signal from labeled tolDCs in the brain of EAE mice. In the present study, only a minority of *in vitro* cultured vitD_3_-treated tolDCs expressed CCR5, despite having a maturation-resistant phenotype (28) and CCR5 being mainly expressed by immature DCs (38). Accordingly, the cells exhibited only marginal chemotaxis to a CCL4 and CCL5 gradient, with less than 0.2% of tolDCs on average displaying chemokine-driven migration. To increase the migratory potential of tolDCs, the cells were transfected with mRNA encoding the CCR5 protein according to a previously optimized protocol for mRNA EP of *in vitro* generated DCs (31, 32). Following mRNA EP, CCR5 protein expression reached its zenith 48 h after EP. Increased CCR5 expression resulted in higher *in vitro* migratory capacity of tolDCs in response to CCL4 and CCL5. Interestingly, mRNA electroporated tolDCs also displayed a higher capacity to transmigrate through the BBB *in vitro* in response to these chemokines. The number of cells needed to achieve a therapeutic effect *in vivo* is not known. Previous research showed that only 2–4% of the total administered population of immune-stimulatory DCs reached lymph nodes following *in vivo* migration but that this low number of cells was sufficient to elicit an antigen-specific immune response *in vivo* (39–42).

The finding that responsiveness of tolDCs to CCR5 ligands can be boosted is of particular relevance for the treatment of MS, as previous studies confirmed that these chemokines were upregulated in the CNS of MS patients (18–22). Moreover, we and others showed that CCR5 ligands were actively transported across the BBB (30, 43, 44). In this way, they provide traffic cues for circulating immune cells to enter the inflamed CNS. Previous research demonstrated that the presence of DCs with tolerogenic properties in the CNS delayed, prevented, or ameliorated EAE (25–27). Therefore, we hypothesize that *CCR5* mRNA-electroporated DCs will outperform nonmodulated tolDCs in terms of efficacy due to their acquired capacity to reach the site of inflammation. However, this hypothesis remains to be tested in *in vivo* models of neuroinflammation.

Besides being implicated in MS pathogenesis, CCR5 ligands drive immune cell accumulation in affected tissue in several other autoinflammatory and immune-mediated diseases. Researchers reported elevated levels of these chemokines in the inflamed synovium of rheumatoid arthritis patients (45), pancreatic islets of type I diabetic patients (46), and intestines of patients with inflammatory bowel disease (47). Hence, modulation of migratory capacity of tolDCs driven by CCR5 may also be advantageous in the treatment of these diseases. The approach described herein can also be applied to enhance the expression of and migration directed by other chemokine receptors, making it possible to tailor the migratory capacity of therapeutically administered cell populations to the chemokine expression profile associated with a specific target organ or disorder. For example, migration of tolDCs to lymph nodes is mainly driven by CCR7. Its ligands, CCL19 and CCL21, are highly expressed by lymph node fibroblastic reticular cells (48–50) and lymphatic endothelial cells (51). They guide mature DCs and specific T-cell subsets to T-cell zones of lymph nodes, coordinating their colocalization for subsequent interaction. CCR7 expression is upregulated on DCs by a maturation stimulus. Likewise, the expression of this chemokine receptor on tolDCs is upregulated after a proinflammatory challenge, albeit expression levels on tolDCs remain significantly lower as compared with those on mature DCs (29, 52, 53). This translates into reduced migratory capacity toward CCL19 and CCL21 *in vitro*. Similarly, introducing CCR7 expression in tolDCs using the proposed approach of chemokine receptor mRNA EP could overcome the limited lymphoid homing capacity of tolDCs.

RNA can act as both a pathogen-associated and damage-associated molecular pattern (54–56). Intracellular introduction of RNA by means of EP could thus lead to DC activation. In agreement with previous findings showing that mature monocyte-derived DCs were not activated by electroporated mRNA (57), we showed that mRNA EP did not affect the semi-mature phenotype, tolerogenic gene expression signature, or allo-stimulatory capacity of vitD_3_-treated tolDCs.

In conclusion, this is the first study to show that enhancing CCR5 expression of tolDCs using mRNA EP endowed these cells with CCR5-driven migratory capacity. This enabled the cells to migrate to inflammatory sites, even when this required crossing of functional barriers, such as the BBB. Importantly, both the tolerogenic phenotype and function of tolDCs were unaffected by the process of mRNA EP. These findings represent an important step forward in the development of a next generation of cell-based tolerance-inducing therapies for the treatment of immune-mediated disorders.

## Author Contributions

All authors have contributed substantially to this work, have approved the manuscript, and agreed with its submission.

## Conflict of Interest Statement

The authors declare that the research was conducted in the absence of any commercial or financial relationships that could be construed as a potential conflict of interest. The reviewer MM and handling editor declared their shared affiliation.
