# Supplementary material for: Shuttling Tolerogenic Dendritic Cells across the Blood–Brain Barrier In Vitro via the Introduction of De Novo C–C Chemokine Receptor 5 Expression Using Messenger RNA Electroporation
Source: Front Immunol. 2018 Jan 23;8:1964. doi: 10.3389/fimmu.2017.01964 (PMC5778265; doi:10.3389/fimmu.2017.01964)
Supplement: Supplementary file 1 [file Table_1.PDF]

Supplementary table 1. Validation data of gene-specific primers

Assay efficiency was determined using a seven-point standard curve from 20 copies to 20 million copies. Ideally, the efficiency equals 100%, representing a perfect doubling of template at every cycle. Typically, good assay efficiencies range between 90-110%. R^2 represents the linearity of the standard curve and how well the standard curve data points fit the linear regression line. Acceptable values are > 0.98. cDNA Cq is the Cq value obtained from 25 ng of cDNA transcribed from universal RNA when performing wet-lab validation of the assay. cDNA Tm is the melting temperature of the amplicon when running a melt curve analysis. gDNA Cq is the Cq value obtained when running the assay with 2.5 ng of genomic DNA, a more than moderate level of genomic DNA contamination. The specificity is represented by the percentage of specific amplicon reads as measured by next generation sequencing (NGS). While 100% specificity is desirable, small decreases in specificity (<1%) can be due to NGS read errors. *Information obtained from Bio-Rad validation reports delivered with gene-specific primers.*

Supplementary table 1.

**Gene symbol**	**Gene name**	**RefSeq Accession No**	**Assay design**	**Efficiency (%)**	**R^2**	**cDNA Cq**	**cDNA Tm (°C)**	**gDNA Cq**	**Specificity (%)**
**LILRB4**	leukocyte immunoglobulin-like receptor, subfamily B (with TM and ITIM domains), member 4	NC_000019.9, NT_011109.16	Intron-spanning	100	0.9995	25.54	87.5	31.99	100
**TLR2**	toll-like receptor 2	NC_000004.11, NG_016229.1, NT_016354.19	Exonic	99	0.9997	25.63	79	25.05	100
**ACTB**	Actin, beta	NC_000007.13, NG_007992.1, NT_007819.17	Exonic	103	0.9939	15.155	82	22.445	/
**PGK1**	phosphoglycerate kinase 1	NC_000023.10, NG_008862.1, NT_011651.17	Exonic	98	0.9996	16.13	81	24.2	100
