# Supplementary material for: Shuttling Tolerogenic Dendritic Cells across the Blood–Brain Barrier In Vitro via the Introduction of De Novo C–C Chemokine Receptor 5 Expression Using Messenger RNA Electroporation
Source: Front Immunol. 2018 Jan 23;8:1964. doi: 10.3389/fimmu.2017.01964 (PMC5778265; doi:10.3389/fimmu.2017.01964)
Supplement: Supplementary file 2 [file Image_1.PDF]

Supplementary Figure 1. Comparison and alignment of the CCR5 gene sequence (Query) and codon-optimized sequence of the CCR5 vector (Sbjct)

```
Query  7762   ATGGATTATCAAGTGTCAAGTCCAATCTATGACATCAATTATTATACATCGGAGCCCTGC  7821
              ||||| || || |||||||| || ||||| |||||||| || || ||    |||||||||
Sbjct  59     ATGGACTACCAGGTGTCAAGCCCCATCTACGACATCAACTACTACACCAGCGAGCCCTGC  118

Query  7822   CAAAAAATCAATGTGAAGCAAATCGCAGCCCGCCTCCTGCCTCCGCTCTACTCACTGGTG  7881
              || |||||||| |||||||| ||||| ||| | || |||||||| || |||   ||||||
Sbjct  119    CAGAAAATCAACGTGAAGCAGATCGCCGCCAGACTGCTGCCTCCTCTGTACAGCCTGGTG  178

Query  7882   TTCATCTTTGGTTTTGTGGGCAACATGCTGGTCATCCTCATCCTGATAAACTGCAAAAGG  7941
              |||||||| || || ||||||||||||||||||||||| |||||||| ||||||||  ||
Sbjct  179    TTCATCTTCGGCTTCGTGGGCAACATGCTGGTCATCCTGATCCTGATCAACTGCAAGCGG  238

Query  7942   CTGAAGAGCATGACTGACATCTACCTGCTCAACCTGGCCATCTCTGACCTGTTTTTCCTT  8001
              ||||||  |||||| |||||||||||||| |||||||| |||   |||||||| |||||
Sbjct  239    CTGAAGTCCATGACCGACATCTACCTGCTGAACCTGGCTATCAGCGACCTGTTCTTCCTG  298

Query  8002   CTTACTGTCCCCTTCTGGGCTCACTATGCTGCCGCCCAGTGGGACTTTGGAAATACAATG  8061
              || || || || || ||||| || ||||| ||||| ||||||||||| || ||||| |||
Sbjct  299    CTGACCGTGCCTTTTTGGGCCCATTATGCCGCCGCTCAGTGGGACTTCGGCAATACCATG  358

Query  8062   TGTCAACTCTTGACAGGGCTCTATTTTATAGGCTTCTTCTCTGGAATCTTCTTCATCATC  8121
              ||||| ||  |||| || || || || || ||||| |||   || |||||||||||||||
Sbjct  359    TGTCAGCTGCTGACTGGCCTGTACTTCATCGGCTTTTTCAGCGGCATCTTCTTCATCATC  418

Query  8122   CTCCTGACAATCGATAGGTACCTGGCTGTCGTCCATGCTGTGTTTGCTTTAAAAGCCAGG  8181
              || || || ||||| || |||||||| || || ||||| ||||||||  | ||||||||
Sbjct  419    CTGCTCACCATCGACAGATACCTGGCCGTGGTGCATGCCGTGTTTGCCCTGAAAGCCAGA  478

Query  8182   ACGGTCACCTTTGGGGTGGTGACAAGTGTGATCACTTGGGTGGTGGCTGTGTTTGCGTCT  8241
              || || |||||||| ||||| ||   |||||| || ||||| || || ||||| || |||
Sbjct  479    ACCGTGACCTTTGGCGTGGTCACCTCTGTGATTACATGGGTCGTCGCCGTGTTCGCCTCT  538

Query  8242   CTCCCAGGAATCATCTTTACCAGATCTCAAAAAGAAGGTCTTCATTACACCTGCAGCTCT  8301
              || || || |||||||| ||||||   || ||||| || || || || ||||||||||||
Sbjct  539    CTGCCTGGCATCATCTTCACCAGAAGCCAGAAAGAGGGCCTGCACTATACCTGCAGCTCT  598

Query  8302   CATTTTCCATACAGTCAGTATCAATTCTGGAAGAATTTCCAGACATTAAAGATAGTCATC  8361
              || || || ||||| ||||| || ||||||||||| || |||||  | ||||| || |||
Sbjct  599    CACTTCCCCTACAGCCAGTACCAGTTCTGGAAGAACTTTCAGACCCTGAAGATCGTGATC  658

Query  8362   TTGGGGCTGGTCCTGCCGCTGCTTGTCATGGTCATCTGCTACTCGGGAATCCTAAAAACT  8421
               |||| || || ||||| ||||| || ||||||||||||||||| || ||||| |||||
Sbjct  659    CTGGGCCTCGTTCTGCCTCTGCTCGTGATGGTCATCTGCTACTCCGGCATCCTGAAAACC  718

Query  8422   CTGCTTCGGTGTCGAAATGAGAAGAAGAGGCACAGGGCTGTGAGGCTTATCTTCACCATC  8481
              ||||| ||||| || || |||||||| || ||||| || ||| |||| |||||||| ||
Sbjct  719    CTGCTGCGGTGCCGGAACGAGAAGAAAAGACACAGAGCCGTGCGGCTGATCTTCACGATT  778

Query  8482   ATGATTGTTTATTTTCTCTTCTGGGCTCCCTACAACATTGTCCTTCTCCTGAACACCTTC  8541
              ||||| || || || || |||||||| || |||||||| || || || ||||||||||||
Sbjct  779    ATGATCGTGTACTTCCTGTTCTGGGCCCCTTACAACATCGTGCTGCTGCTGAACACCTTC  838

Query  8542   CAGGAATTCTTTGGCCTGAATAATTGCAGTAGCTCTAACAGGTTGGACCAAGCTATGCAG  8601
              || || ||||| |||||||| || |||   |||   |||||  ||||||| || |||||
Sbjct  839    CAAGAGTTCTTCGGCCTGAACAACTGCTCCAGCAGCAACAGACTGGACCAGGCCATGCAA  898

Query  8602   GTGACAGAGACTCTTGGGATGACGCACTGCTGCATCAACCCCATCATCTATGCCTTTGTC  8661
              ||||| ||||| || || ||||| |||||||||||||||||||||||||| ||||||||
Sbjct  899    GTGACCGAGACACTGGGCATGACCCACTGCTGCATCAACCCCATCATCTACGCCTTTGTG  958

Query  8662   GGGGAGAAGTTCAGAAACTACCTCTTAGTCTTCTTCCAAAAGCACATTGCCAAACGCTTC  8721
              || ||||||||| | || |||||  | || ||||| || |||||||||||||| || |||
Sbjct  959    GGCGAGAAGTTCCGGAATTACCTGCTGGTGTTCTTTCAGAAGCACATTGCCAAGCGGTTC  1018

Query  8722   TGCAAATGCTGTTCTATTTTCCAGCAAGAGGCTCCCGAGCGAGCAAGCTCAGTTTACACC  8781
              ||||| ||||| || || || ||||||||||| || ||  | || |||   || |||||
Sbjct  1019   TGCAAGTGCTGCTCCATCTTTCAGCAAGAGGCCCCTGAAAGGGCCAGCAGCGTGTACACA  1078

Query  8782   CGATCCACTGGGGAGCAGGAAATATCTGTGGGCTTGTGAC  8821
               ||  ||| || ||||| || ||    |||||  ||| ||
Sbjct  1079   AGAAGCACCGGCGAGCAAGAGATCAGCGTGGGACTGTAAC  1118
```
